# Omega-3 polyunsaturated fatty acids supplementation in patients with diabetes and cardiovascular disease risk: does dose really matter?

**DOI:** 10.1186/s12933-018-0766-0

**Published:** 2018-08-28

**Authors:** Alexander Tenenbaum, Enrique Z. Fisman

**Affiliations:** 1 Sackler Faculty of Medicine, Tel Aviv University, 6997801 Tel Aviv, Israel; 2 Cardiac Rehabilitation Institute, Sheba Medical Center, 5265601 Tel Hashomer, Israel

**Keywords:** Atherogenesis, Cardiovascular risk reduction, Coronary artery disease, Dyslipidemia, Hypertriglyceridemia, Omega 3 supplements, Polyunsaturated fatty acids, Type 2 diabetes mellitus

## Abstract

There is a vast disagreement in relation to the possible beneficial effects of omega-3 polyunsaturated fatty acids (omega-3 PUFA) supplementation in patients with diabetes and cardiovascular disease. The conflicting results between the various original studies and meta-analyses could be partially explained as a result of variable supplementation dosage and duration, either of which may modify the effects of omega-3 PUFA on cardio-metabolic biomarkers. Meta-analyses are limited usually by the inability to draw inferences regarding dosage, duration and the interaction of dosage and duration of omega-3 PUFA intake. Even so, almost all endpoints in the so-called “negative” meta-analyses leaned toward a trend for benefit with a near 10% reduction in cardiovascular outcomes and a borderline statistical significance. Many trials included in these meta-analyses tested an insufficient daily dose of omega-3 PUFA of less than 1000 mg. Probably, the consistent cardiovascular effects of omega-3 PUFA supplements could be expected only with daily doses above 2000 mg.

## Main text

There is a vast disagreement in relation to the possible beneficial effects of omega-3 polyunsaturated fatty acids (omega-3 PUFA) supplementation in patients with diabetes and cardiovascular disease. The divergences have been just fueled by two recently published meta-analyses establishing that omega-3 PUFA supplementation may not reduce the risk for cardiovascular events [1, 2]. These negative conclusions are accompanied by a number of studies demonstrating no benefits from omega-3 PUFA supplementation on oxidative stress, inflammatory parameters, coagulation and metabolic status in patients with atherosclerotic vascular disease and type 2 diabetes mellitus (T2DM) [3, 4].

In sharp contrast, the largest, most comprehensive and contemporary meta-analysis of randomized controlled trials has shown that omega-3 PUFA supplementation produces favorable hypolipidemic effects, a reduction in pro-inflammatory cytokine levels and improvement in glycemia [5]. The positive conclusions are complemented by an impressive group of most of the latest studies, demonstrating beneficial effects of omega-3 PUFA supplementation on metabolism [6–9]. Sawada et al. validated the fact that one of the omega-3 PUFAs—eicosapentaenoic acid—corrected postprandial hypertriglyceridemia, hyperglycemia and insulin secretion ability. This amelioration of several metabolic abnormalities was accompanied by recovery of concomitant endothelial dysfunction in patients with impaired glucose metabolism and coronary artery disease (CAD) [6]. In patients with residual hypertriglyceridemia despite statin treatment, a combination of omega-3 PUFA and rosuvastatin produced a greater reduction of triglyceride and non-HDL-cholesterol than rosuvastatin alone [7]. Omega-3 PUFA supplementation attenuated the progression of albuminuria in subjects with T2DM and CAD [8].

Jacobo-Cejudo et al. found a beneficial effect of omega-3 PUFA supplementation on waist circumference, glucose, Hb1Ac, leptin and leptin/adiponectin ratio [9]. It is well established that T2DM is associated with hypertriglyceridemia as a major component of atherogenic dyslipidemia, which significantly increases cardiovascular disease risk [10–12]. Specifically, hepatic insulin resistance in T2DM patients is the main determinant of postprandial lipoprotein metabolism and hypertriglyceridemia [13]. Omega-3 PUFA intake has been widely indicated for treatment of hypertriglyceridemia, promoting reductions in hepatic triglyceride synthesis and accelerating triglyceride clearance [14–17]. The benefits of omega-3 PUFA intake in appropriate doses (above 2000 mg daily) on serum triglyceride levels are well-documented and not a matter of debate. Theoretically, these favourable effects have a pathophysiological basis to be translated into cardiovascular benefits.

The conflicting results between the various original studies and meta-analyses could be partially explained as a result of variable supplementation dosage and duration, either of which may modify the effects of omega-3 PUFA on cardio-metabolic biomarkers [18]. Meta-analyses are limited usually by the inability to draw inferences regarding dosage, duration and the interaction of dosage and duration of omega-3 PUFA intake. Even so, almost all endpoints in the so-called “negative” meta-analyses leaned toward a trend for benefit with a near 10% reduction in cardiovascular outcomes and a borderline statistical significance. Many trials included in these meta-analyses tested an insufficient daily dose of omega-3 PUFA of less than 1000 mg. Probably, the consistent cardiovascular effects of omega-3 PUFA supplements could be expected only with daily doses above 2000 mg.

Hopefully, two ongoing randomized controlled trials, REDUCE-IT and STRENGTH, which are assessing a daily dose of omega-3 PUFA of 4000 mg in addition to statins in patients with hypertriglyceridemia, have potential for a soon clarification of the current controversies [19].

## Abbreviations

CAD: coronary artery disease
PUFA: polyunsaturated fatty acids
T2DM: type 2 diabetes mellitus
